# *In vitro* evaluation of β-carboline alkaloids as potential anti-*Toxoplasma* agents

**DOI:** 10.1186/1756-0500-6-193

**Published:** 2013-05-10

**Authors:** Maria L Alomar, Federico AO Rasse-Suriani, Agustina Ganuza, Verónica M Cóceres, Franco M Cabrerizo, Sergio O Angel

**Affiliations:** 1 Laboratorio de Parasitología Molecular, IIB-INTECH, CONICET/UNSAM, Av. Intendente Marino Km. 8.2, C.C 164, (B7130IIWA) Chascomús, Prov. Buenos Aires, Argentina; 2 Laboratorio de Fotoquímica y Fotobiología Molecular, IIB-INTECH, CONICET/UNSAM, Av. Intendente Marino Km. 8.2, C.C 164, (B7130IIWA) Chascomús, Prov. Buenos Aires, Argentina

**Keywords:** *Toxoplasma gondii*, β-carbolines, Drug, Invasion, Cell cycle

## Abstract

**Background:**

Toxoplasmosis is a worldwide infection caused by the protozoan parasite *Toxoplasma gondii*, which causes chorioretinitis and neurological defects in congenitally infected newborns or immunodeficient patients. The efficacy of the current treatment is limited, primarily by serious host toxicity. In recent years, research has focused on the development of new drugs against *T. gondii*. β-Carbolines (βCs), such as harmane, norharmane and harmine, are a group of naturally occurring alkaloids that show microbicidal activity. In this work, harmane, norharmane and harmine were tested against *T. gondii*.

**Findings:**

The treatment of extracellular tachyzoites with harmane, norharmane and harmine showed a 2.5 to 3.5-fold decrease in the invasion rates at doses of 40 μM (harmane and harmine) and 2.5 μM (norharmane) compared with the untreated parasites. Furthermore, an effect on the replication rate could also be observed with a decrease of 1 (harmane) and 2 (norharmane and harmine) division rounds at doses of 5 to 12.5 μM. In addition, the treated parasites presented either delayed or no monolayer lysis compared with the untreated parasites.

**Conclusions:**

The three βC alkaloids studied (norharmane, harmane and harmine) exhibit anti-*T. gondii* effects as evidenced by the partial inhibition of parasite invasion and replication. A dose–response effect was observed at a relatively low drug concentration (< 40 μM), at which no cytotoxic effect was observed on the host cell line (Vero).

## Findings

The protozoan parasite *Toxoplasma gondii* is the etiologic agent of toxoplasmosis, a worldwide infection affecting 500 million to 1 billion people [1]. Toxoplasmosis occurs as an asymptomatic chronic (latent) infection. However, it is of medical health importance because toxoplasmosis can be dangerous or even fatal in immunocompromised individuals because of the reactivation of a latent infection. Congenital infection with *Toxoplasma* can also cause either spontaneous abortion or birth defects [2]. In the latter case, the active form of the parasite can cause encephalitis and neurologic diseases and can affect the heart, liver, inner ears, and eyes (chorioretinitis). Recently, chronic toxoplasmosis has been linked with brain cancer, attention deficit hyperactivity disorder, obsessive-compulsive disorder and schizophrenia [3-6].

There are effective drug regimens for toxoplasmosis based on a combination of pyrimethamine and sulfadiazine, but in some cases the efficacy of the current treatment is limited, primarily by serious host toxicity and/or development of drug-resistant parasites. In patients under immunosuppressive therapies and particularly in those with AIDS, treatment with sulfonamides and inhibitors of dihydrofolate reductase (DHFR) can produce side effects despite the preventive administration of folinic acid [7,8]. Moreover, the current therapy is ineffective against tissue cysts [9]. Other therapies, based on other types of drugs (clindamycin, spiramycin or atovaquone), have been used with limited success particularly in long-term patient management.

**Figure 1:**
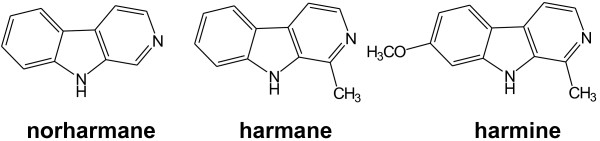
Structure of the different βCs studied: harmane, norharmane and harmine.

In recent years, research has focused on the development of new drugs against *T. gondii*[10-12]. Nevertheless, the need to identify and evaluate new drugs based on new and innovative therapeutic strategies against *T. gondii* is still evident. In this regard, β-carbolines (βCs) represent an excellent alternative that should be thoroughly investigated. βCs comprise a class of alkaloids that are widely found in nature. These alkaloids normally occur in plants [13,14] and mammals [15-17]. βCs are a group of heterocyclic compounds with a 9H-pyrido[3,4-b]indole structural unit (Figure 1) [18].

βCs were originally isolated from *Peganumharmala* (*Zygophyllaceae*, Syrian Rue), which is used as a traditional herbal drug [19,20]. Among them, harmane and tetrahydroharmane isolated from active extracts of different plants have been shown to have antimalarial activity and low cytotoxicity for human cells [21-23]. In addition, harmane and harmine have been shown a moderate effect on promastigotes of *Leishmania infantum*, mainly by accumulation of parasites arrested in the S-G2/M phases of the cell cycle, whereas harmaline has shown only anti-leishmanial activity against intracellular amastigotes by inhibiting the PKC enzyme [24]. Lala *et al*. [25] have shown that harmine is toxic for the promastigotes of *Leishmania donovani*, an effect attributed to necrosis due to non-specific membrane damage. The nifurtimox-resistant *Trypanosoma cruzi* LQ strain has shown a greater sensitivity to βCs, most likely due to inhibition of the respiratory chain of the parasite [26]. The chaperone HSP90 is an important drug target in protozoan parasites and new drugs are actively being sought [12,27]. In this regard, harmine was one of the three compounds selected among approximately 4,000 small molecules that inhibited *P. falciparum* HSP90 by specific competition with its ATP-binding domain. Interestingly, harmine has been shown to have anti-malarial effects *in vitro* and *in vivo* and acts synergistically with chloroquine and artemisinin [28,29]. El Sayed *et al. *[30] also reported that new enantiomers of 8-hydroxymanzamine A (ent-8-hydroxymanzamine A) and manzamine F (entmanzamine), isolated from Indo-Pacific sponge, together with manzamine A, exhibit significant activities against *T. gondii* and *Plasmodium* spp. There are also other studies in which βCs have shown parasiticidal and microbicidal effects that were further revised by Cao *et al. *[20].

The success of *T. gondii* infection relies on host cell recognition and attachment, invasion, replication and egress to spread throughout the host organism. Therefore, blocking any of these processes represents a target for new anti-*Toxoplasma* drugs/therapies. Our laboratory has gained experience in the analysis of harmane, norharmane and harmine for different purposes including their use as anti-viral drugs (Cabrerizo *et al*., unpublished results). The biological and pharmacological effects of these βCs are attributed in part to their ability to intercalate DNA and inhibit topoisomerase I and II, effects that result in alterations in DNA replication and, therefore, defects in cell cycle progress [20]. In addition, harmine has been found to be a potent and specific inhibitor of cyclin-dependent kinases (CDKs), showing a strong inhibitory effect on the growth and proliferation of carcinoma cells [31,32]. In this work, we evaluated these three βC alkaloids, harmane, norharmane and harmine, for their potential as new drugs against *T. gondii*, based on their ability to block invasion, replication and growth processes.

## Materials and methods

### Chemicals

Norharmane, harmane, harmine and sulfadiazine (Sigma-Aldrich Co., Buenos Aires, Argentina) were of the highest purity available (> 98%) and used without further purification.

### Stock solutions preparation and pH adjustment

βC stock solutions (approximately 2 mM) were prepared as described elsewhere [33]. Briefly, each alkaloid was dissolved in acid-sterilized water. Once the alkaloid was fully dissolved, the pH of the aqueous solutions was adjusted to 7.4 by the addition of drops of HCl or NaOH solutions from a micropipette. The aliquots used in the *in vitro* experiments did not modify the pH of the cell culture. Sulfadiazine was dissolved in dimethyl sulfoxide (DMSO, Sigma-Aldrich Co.) at 22 mM.

### Parasite sources, culture and manipulation

Tachyzoites of the RH strain were cultured in standard tachyzoite conditions *in vitro*: Vero and human foreskin fibroblast (HFF) cell monolayers were infected with tachyzoites and incubated with Dulbecco’s modified Eagle medium (DMEM, Gibco BRL) supplemented with 10% fetal bovine serum (FBS), penicillin (100 UI/ml; Gibco BRL) and streptomycin (100 μg/ml; Gibco BRL) at 37°C and 5% CO_2_.

### Invasion

Tachyzoites of the RH strain (2.5×10^6^) were incubated for 1 h with different doses of βCs or water (untreated) in DMEM at 37°C. Treated and untreated tachyzoites were added to a Vero cell monolayer, incubated for 10 min on ice and then incubated at 37°C for 1 h, washed three times with PBS and fixed with 4% paraformaldehyde and 0.2% Triton X-100 and analyzed by immunofluorescence using murine anti-SAG1 antibody (SAG1: *T. gondii* surface antigen 1, 1:100 dilution)/Alexa Fluor 594-conjugated goat anti-mouse (1:4,000 dilution; Invitrogen, Argentina). In each experiment, 50 fields were analyzed and the number of tachyzoites per field was counted. Only fields with a similar number of host cells were considered for the experiments. The mean numbers of tachyzoites in each field plus the standard deviation were plotted using Prism GraphPad software.

### Replication and growth analysis

The replication rate was determined by incubating the tachyzoites (2.5×10^6^) with Vero cell monolayers, followed by 10 min of incubation on ice, then 1 h at 37°C and washing three times with PBS. Finally, the tachyzoites were incubated for an additional 24 h at 37°C, 5% CO_2_ with DMEM, 5% FBS and different doses of βCs or water (untreated). After the infected monolayers were fixed, they were immunolabeled with murine anti-SAG1 antibody/Alexa Fluor 594-conjugated goat anti-mouse antibody (Invitrogen, Argentina) and the number of tachyzoites per parasitophorous vacuole (PV) were counted. Fifty fields were counted in duplicate. Approximately 1000 PVs were counted at each dose. Data are presented as a percentage of PVs that contained a geometric progression (e.g. 1, 2, 4, 8 and so on) of tachyzoites per PV.

To analyze parasite growth, human foreskin fibroblast (HFF) monolayers were incubated with 2×10^4^ tachyzoites as above mentioned. Because tachyzoite growth is destructive to cell monolayers, infected cultures were followed daily by inverted microscope visualization until complete monolayer lysis along with the presence of extracellular tachyzoites or complete (host cell/tachyzoites) destruction.

## Results and discussion

**Figure 2:**
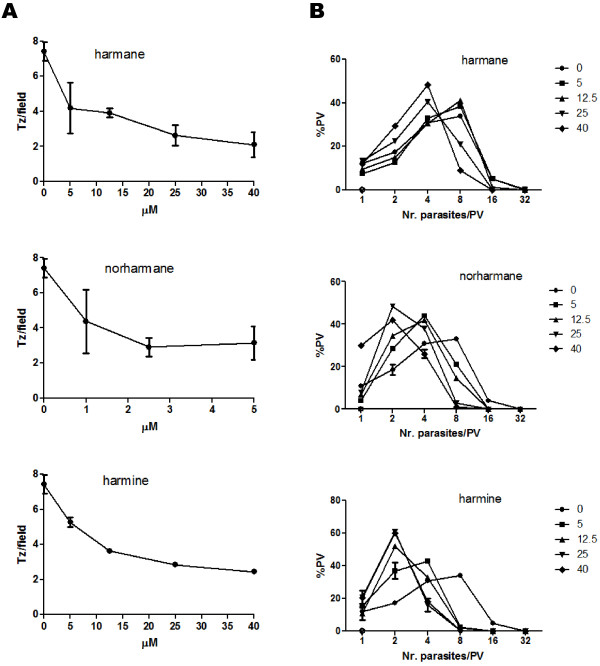
**Effects of harmane, norharmane and harmine on parasite invasion and replication. A**. Extracellular tachyzoites (2.5×10^6^) were treated for 1 h with different doses of the βCs, washed and allowed to invade Vero cells monolayers for 1 h at 37°C, and then fixed and incubated with murine anti-SAG1 antibody. The secondary antibody used was Alexa Fluor 594 goat anti-rabbit. For each treatment, the number of parasitophorous vacuoles (PVs) per field was counted. Fifty fields selected at random were analyzed in duplicate. **B.** Infected monolayers were treated for 24 h with different doses of the βCs, fixed and immunostained with anti-SAG1 antibody. The number of tachyzoites inside the PV was counted. Fifty fields selected at random were counted in duplicate. In total, approximately 1000 PVs per dose were analyzed in **A** and **B**. These panels are representative of three independent experiments. All the experiments presented similar results.

To analyze the effect of harmane, norharmane and harmine alkaloids on the ability of *T. gondii* to infect host cells, 2.5×10^6^ extracellular tachyzoites (almost all comprising a G1 arrested form of the parasite) were incubated for 1 h with different doses (0 to 100 μM) of each βC. This dose range was chosen because it was previously shown that βCs and especially harmine induce apoptosis and necrosis, and inhibit proliferation of eukaryotic cells at concentrations higher than 40 μM [34]. Moreover, for some eukaryotic cell lines, such concentrations are cytotoxic [32]. Therefore, the selected βC-concentration range was chosen to avoid any secondary effect on the host cell line (Vero) that could interfere with the analysis of the invasion capability of the tachyzoites. The results plotted in Figure 2 (A) show that the three βCs studied have an inhibitory effect on parasite invasion in a dose-dependent manner. Norharmane exhibited its maximum effect at 2.5 μM whereas harmane and harmine had the highest parasiticidal effect at 40 μM (Figure 2A). The invasion rate of tachyzoites treated with βCs at the doses mentioned before showed a 3.5- (harmane), 2.5- (norharmane) and 3.1- (harmine) fold decrease compared with the untreated tachyzoites. Because this assay does not differentiate if the components affect the invasion process of the tachyzoites or the attachment process, we can only conclude that the decrease in the number of tachyzoites per field could be caused by an inhibitory effect on invasion, or attachment, or both.

To determine whether these βCs have any inhibitory effect on the parasite cycle process, we measured the ability of intracellular-treated parasites to replicate. Because tachyzoites replicate only within the host cell by a process called endodyogeny, every round of replication results in a geometric duplication of the parasite number per PV (e.g. 1, 2, 4, 8 and so on) [35]. Monolayers of Vero cells were infected with 2.5×10^6^ of fresh tachyzoites to allow for the formation of PVs. Subsequently, the infected monolayers were washed and incubated at 37°C for 24 h in the presence of different doses of βCs (0 to 40 μM) (Figure 2B). Every PV was generated from one parasite; therefore, the number of tachyzoites inside the PV indicates the number of replication events. The panel shows the percentage of PVs that presented different numbers of tachyzoites. Harmane showed a slight inhibitory effect starting at 25 μM, resulting in a delay of one round of replication compared with the untreated parasites. Harmine and norharmane had a stronger inhibitory effect on parasite replication than harmane, because their effect was more pronounced at 25 μM, at which concentration the replication rate was delayed by two rounds (Figure 2B). Doses of norharmane lower than 5 μM did not show any effect on parasite replication (Additional file 1: Figure S1).

**Figure 3:**
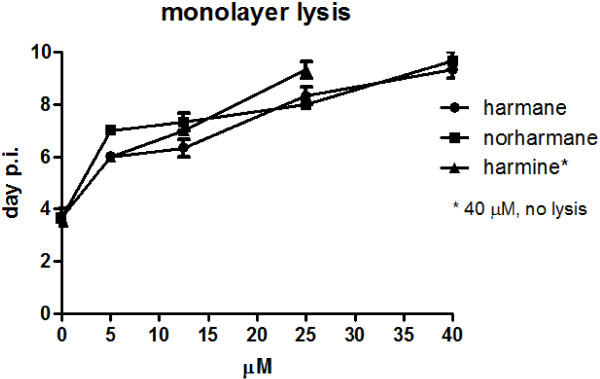
**Analysis of the toxic effect of harmane, norharmane and harmine on *T. gondii *growth.** Human foreskin fibroblast (HFF) monolayers were infected with fresh tachyzoites (2.5×10^4^) for 1 h and incubated with different doses of βCs and followed by microscope observation until monolayer lysis (cell lysis in combination with extracellular tachyzoites). Data are presented as the day post-infection (p.i.) on which complete monolayer lysis was observed by microscope visualization. The panel is representative of three independent experiments performed in duplicate. All the experiments presented similar results. Sulfadiazine (5 mM) was used as a control of *T. gondii* growth inhibitory drugs and showed no monolayer lysis at day 10 p.i.

*Toxoplasma gondii* growth is the result of repeated cycles of host cell invasion, replication and egress, resulting in cell monolayer destruction *in vitro*. Because we observed a low rate of parasite entry and tachyzoite replication in the host cell, we expected a low rate of parasite growth. To analyze for this effect, HFF monolayers were infected with fresh tachyzoites, washed and incubated with the different doses of the βCs until the complete monolayer was lysed. HFF monolayers were completely lysed at day 3–4 after infection with the untreated parasites. For βC-treated monolayers, a delay in the time of complete lysis could be observed (Figure 3). As shown in Figure 3, harmine was shown to affect parasite growth at 40 μM. Infected monolayers treated with 5 mM of sulfadiazine did not show cell lysis during the 10 days of incubation (data not shown).

Our results demonstrate that the three βCs studied in this work have anti-*T. gondii* effects on both host cell invasion and replication processes and, consequently, on parasite growth. Particularly, harmine was shown to be the most active drug because it showed the strongest inhibitory effect on parasite replication and growth. In a recent study [25], it has been observed that harmine shows anti-leishmanial activity in part due to cell death attributed to non-specific membrane damage. In our hands, the highest dose (40 μM) of βCs affected neither the cell integrity nor the viability of the parasite. The entry of the tachyzoite into the host cell is an ATP-consuming process that includes the glideosome/myosin motor [36]. In this sense, some βCs have shown an inhibitory effect of the respiratory chain [26,37]. It is possible that parasite fitness is decreased because of a failure of mitochondrial functions. Our analysis did not allow us to distinguish which process (es) these drugs were affecting (attachment and/or invasion). Therefore, the βCs could be interfering with several metabolism pathways other than those related to attachment/invasion of the host cell, resulting in an impairment of these processes. Further analysis should be performed to elucidate which process/es is/are involved.

As mentioned above, βCs are considered to affect cell cycle. The tachyzoite is the highly replicative stage of *T. gondii*, a process that only occurs inside the host cell [38]. It is well known that these drugs can bind to DNA [39] and induce DNA damage [40] as well as inhibit topoisomerases I and II. These facts can contribute to the replication-associated DNA stress, affecting the cell replication rate [41,42]. Harmine also affects *Plasmodium* infection through its interaction with parasite HSP90, a recognized drug target against malaria [12,29]. The effect of this (or these) βC(s) on parasite replication could also involve the inhibition of *Toxoplasma* HSP90 functions. In fact, *T. gondii* HSP90 has been suggested as a key molecule in parasite replication [43]. Future studies should be performed to assess the interaction between these alkaloids and *T. gondii* topoisomerases and/or HSP90 protein and evaluate if these drugs can affect parasite cell cycle progression.

In conclusion, we have demonstrated that the three βC alkaloids studied (norharmane, harmane and harmine) exhibit anti-*T. gondii* effects. Interestingly, the effect on parasite invasion and replication occurred at low doses (below 40 μM), at which these alkaloids did not show any toxic effects on Vero and HFF cells used in our experiments [32,34]. However, βCs have been shown to inhibit some enzymes associated with mental disorders and could produce behavioral modifications, including hallucinogens in treated people [20]. This issue should be considered to determine *in vivo* doses [44]. Moreover, on the basis of the current knowledge [44], it would be possible to design novel harmine derivatives to avoid collateral effects without changing or even increasing the parasiticidal effect. Future studies should be performed to further elucidate the mechanism for their anti-*T. gondii* activity.

## Competing interests

The authors declare that no conflicts of interest exist.

## Authors’ contributions

MLA, conceived the study, participated in its design, performed the laboratory work and drafted the manuscript. FMC and SOA conceived the study, participated in its design and drafted the manuscript. FAORS collaborated with the cell viability and invasion assays. AG collaborated with the replication and growth assays. MVC participated in the experimental design and analysis of the data. All authors have read and approved the final manuscript.

## Supplementary Material

Additional file 1: Figure S1 Effect of norharmane on parasite invasion and replication. The analysis was similar to that mentioned in the Figure 3 legend, except that the drug doses were 0, 1, 2.5 and 5 μM. Click here for file

## References

[B1] Tenter AM Heckeroth AR Weiss LM Toxoplasma gondii: from animals to humans Int J Parasitol 2000 30 12–13 1217 1258 11113252 10.1016/s0020-7519(00)00124-7 PMC3109627

[B2] Carlier Y Truyens C Deloron P Peyron F Congenital parasitic infections: a review Acta Trop 2012 121 2 55 70 10.1016/j.actatropica.2011.10.018 22085916

[B3] Brynska A Tomaszewicz-Libudzic E Wolanczyk T Obsessive-compulsive disorder and acquired toxoplasmosis in two children Eur Child Adolesc Psychiatry 2001 10 3 200 204 10.1007/s007870170027 11596821

[B4] Miman O Mutlu EA Ozcan O Atambay M Karlidag R Unal S Is there any role of Toxoplasma gondii in the etiology of obsessive-compulsive disorder? Psychiatry Res 2010 177 1–2 263 265 20106536 10.1016/j.psychres.2009.12.013

[B5] Yolken RH Dickerson FB Fuller Torrey E Toxoplasma and schizophrenia Parasite Immunol 2009 31 11 706 715 10.1111/j.1365-3024.2009.01131.x 19825110

[B6] Vittecoq M Elguero E Lafferty KD Roche B Brodeur J Gauthier-Clerc M Misse D Thomas F Brain cancer mortality rates increase with Toxoplasma gondii seroprevalence in France Infect Genet Evol 2012 12 2 496 498 10.1016/j.meegid.2012.01.013 22285308

[B7] Luft BJ Remington JS Toxoplasmic encephalitis in AIDS Clin Infect Dis 1992 15 2 211 222 10.1093/clinids/15.2.211 1520757

[B8] Wong SY Remington JS Biology of Toxoplasma gondii AIDS 1993 7 3 299 316 10.1097/00002030-199303000-00001 8471191

[B9] Weiss LM Kim K The development and biology of bradyzoites of Toxoplasma gondii Front Biosci 2000 5 D391 405 10.2741/Weiss 10762601 PMC3109641

[B10] Rodriguez JB Szajnman SH New antibacterials for the treatment of toxoplasmosis; a patent review Expert Opin Ther Pat 2012 22 3 311 333 10.1517/13543776.2012.668886 22404108

[B11] Vanagas L Jeffers V Bogado SS Dalmasso MC Sullivan WJ Jr Angel SO Toxoplasma histone acetylation remodelers as novel drug targets Expert Rev Anti Infect Ther 2012 10 10 1189 1201 10.1586/eri.12.100 23199404 PMC3581047

[B12] Angel SO Matrajt M Echeverria PC A review of recent patents on the protozoan parasite HSP90 as a drug target 2012 Biotechnol: Recent Pat 10.2174/1872208311307010002 PMC3706948 23002958

[B13] Herraiz T Gonzalez D Ancin-Azpilicueta C Aran VJ Guillen H: beta-Carboline alkaloids in Peganum harmala and inhibition of human monoamine oxidase (MAO) Food Chem Toxicol 2010 48 3 839 845 10.1016/j.fct.2009.12.019 20036304

[B14] Pfau W Skog K Exposure to beta-carbolines norharman and harman J Chromatogr B Analyt Technol Biomed Life Sci 2004 802 1 115 126 10.1016/j.jchromb.2003.10.044 15036003

[B15] Pari K Sundari CS Chandani S Balasubramanian D: beta-carbolines that accumulate in human tissues may serve a protective role against oxidative stress J Biol Chem 2000 275 4 2455 2462 10.1074/jbc.275.4.2455 10644699

[B16] Spijkerman R van den Eijnden R van de Mheen D Bongers I Fekkes D The impact of smoking and drinking on plasma levels of norharman Eur Neuropsychopharmacol 2002 12 1 61 71 10.1016/S0924-977X(01)00143-2 11788242

[B17] Torreilles J Guerin MC Previero A Simple compounds with high pharmacologic potential: beta-carbolines. Origins, syntheses, biological properties Biochimie 1985 67 9 929 947 10.1016/S0300-9084(85)80289-3 3910114

[B18] Gonzalez MM Arnbjerg J Denofrio MP Erra-Balsells R Ogilby PR Cabrerizo FM One- and two-photon excitation of beta-carbolines in aqueous solution: pH-dependent spectroscopy, photochemistry, and photophysics J Phys Chem A 2009 113 24 6648 6656 10.1021/jp902105x 19514786

[B19] Sobhani AM Ebrahimi SA Mahmoudian M An in vitro evaluation of human DNA topoisomerase I inhibition by Peganum harmala L. seeds extract and its beta-carboline alkaloids J Pharm Pharm Sci 2002 5 1 19 23 12042115

[B20] Cao R Peng W Wang Z Xu A beta-Carboline alkaloids: biochemical and pharmacological functions Curr Med Chem 2007 14 4 479 500 10.2174/092986707779940998 17305548

[B21] Azas N Laurencin N Delmas F Di GC Gasquet M Laget M Timon-David P Synergistic in vitro antimalarial activity of plant extracts used as traditional herbal remedies in Mali Parasitol Res 2002 88 2 165 171 10.1007/s004360100454 11936507

[B22] Ancolio C Azas N Mahiou V Ollivier E Di Giorgio C Keita A Timon-David P Balansard G Antimalarial activity of extracts and alkaloids isolated from six plants used in traditional medicine in Mali and Sao Tome Phytother Res 2002 16 7 646 649 10.1002/ptr.1025 12410545

[B23] Fiot J Jansen O Akhmedjanova V Angenot L Balansard G Ollivier E HPLC quantification of alkaloids from Haplophyllum extracts and comparison with their cytotoxic properties Phytochem Anal 2006 17 5 365 369 10.1002/pca.927 17019939

[B24] Di Giorgio C Delmas F Ollivier E Elias R Balansard G Timon-David P In vitro activity of the beta-carboline alkaloids harmane, harmine, and harmaline toward parasites of the species Leishmania infantum Exp Parasitol 2004 106 3–4 67 74 15172213 10.1016/j.exppara.2004.04.002

[B25] Lala S Pramanick S Mukhopadhyay S Bandyopadhyay S Basu MK Harmine: evaluation of its antileishmanial properties in various vesicular delivery systems J Drug Target 2004 12 3 165 175 10.1080/10611860410001712696 15203896

[B26] Rivas P Cassels BK Morello A Repetto Y Effects of some beta-carboline alkaloids on intact Trypanosoma cruzi epimastigotes Comp Biochem Physiol C Pharmacol Toxicol Endocrinol 1999 122 1 27 31 10.1016/S0742-8413(98)10069-5 10190025

[B27] Rochani AK Singh M Tatu U Heat Shock Protein 90 Inhibitors as Broad Spectrum Anti-infectives Curr Pharm Des 2012 10.2174/138161213804143608 22920905

[B28] Shahinas D Liang M Datti A Pillai DR A repurposing strategy identifies novel synergistic inhibitors of Plasmodium falciparum heat shock protein 90 J Med Chem 2010 53 9 3552 3557 10.1021/jm901796s 20349996

[B29] Shahinas D Macmullin G Benedict C Crandall I Pillai DR Harmine is a potent antimalarial targeting Hsp90 and synergizes with chloroquine and artemisinin Antimicrob Agents Chemother 2012 56 8 4207 4213 10.1128/AAC.00328-12 22615284 PMC3421604

[B30] El Sayed KA Kelly M Kara UA Ang KK Katsuyama I Dunbar DC Khan AA Hamann MT New manzamine alkaloids with potent activity against infectious diseases J Am Chem Soc 2001 123 9 1804 1808 10.1021/ja002073o 11456797

[B31] Song Y Wang J Teng SF Kesuma D Deng Y Duan J Wang JH Qi RZ Sim MM β-Carbolines as specific inhibitors of cyclin-Dependent kinases Bioorg Med Chem Lett 2002 12 7 1129 1132 10.1016/S0960-894X(02)00094-X 11909733

[B32] Song Y Kesuma D Wang J Deng Y Duan J Wang JH Qi RZ Specific inhibition of cyclin-dependent kinases and cell proliferation by harmine Biochem Biophys Res Commun 2004 317 1 128 132 10.1016/j.bbrc.2004.03.019 15047157

[B33] Gonzalez MM Salum ML Gholipour Y Cabrerizo FM Erra-Balsells R Photochemistry of norharmane in aqueous solution Photochem Photobiol Sci 2009 8 8 1139 1149 10.1039/b822173a 19639116

[B34] Jimenez J Riveron-Negrete L Abdullaev F Espinosa-Aguirre J Rodriguez-Arnaiz R Cytotoxicity of the beta-carboline alkaloids harmine and harmaline in human cell assays in vitro Exp Toxicol Pathol 2008 60 4–5 381 389 18430551 10.1016/j.etp.2007.12.003

[B35] Striepen B Jordan CN Reiff S van Dooren GG Building the perfect parasite: cell division in apicomplexa PLoS Pathog 2007 3 6 e78 10.1371/journal.ppat.0030078 17604449 PMC1904476

[B36] Baum J Papenfuss AT Baum B Speed TP Cowman AF Regulation of apicomplexan actin-based motility Nat Rev Microbiol 2006 4 8 621 628 10.1038/nrmicro1465 16845432

[B37] Bringmann G Feineis D Bruckner R Blank M Peters K Peters EM Reichmann H Janetzky B Grote C Clement HW Bromal-derived tetrahydro-beta-carbolines as neurotoxic agents: chemistry, impairment of the dopamine metabolism, and inhibitory effects on mitochondrial respiration Bioorg Med Chem 2000 8 6 1467 1478 10.1016/S0968-0896(00)00073-0 10896123

[B38] Radke JR Striepen B Guerini MN Jerome ME Roos DS White MW Defining the cell cycle for the tachyzoite stage of Toxoplasma gondii Mol Biochem Parasitol 2001 115 2 165 175 10.1016/S0166-6851(01)00284-5 11420103

[B39] Gonzalez MM Vignoni M Pellon-Maison M Ales-Gandolfo MA Gonzalez-Baro MR Erra-Balsells R Epe B Cabrerizo FM Photosensitization of DNA by beta-carbolines: kinetic analysis and photoproduct characterization Org Biomol Chem 2012 10 9 1807 1819 10.1039/c2ob06505c 22249177

[B40] Gonzalez MM Pellon-Maison M Ales-Gandolfo MA Gonzalez-Baro MR Erra-Balsells R Cabrerizo FM Photosensitized cleavage of plasmidic DNA by norharmane, a naturally occurring beta-carboline Org Biomol Chem 2010 8 11 2543 2552 10.1039/c002235g 20499453

[B41] Hsiang YH Lihou MG Liu LF Arrest of replication forks by drug-stabilized topoisomerase I-DNA cleavable complexes as a mechanism of cell killing by camptothecin Cancer Res 1989 49 18 5077 5082 2548710

[B42] D'Arpa P Beardmore C Liu LF Involvement of nucleic acid synthesis in cell killing mechanisms of topoisomerase poisons Cancer Res 1990 50 21 6919 6924 1698546

[B43] Echeverria PC Matrajt M Harb OS Zappia MP Costas MA Roos DS Dubremetz JF Angel SO Toxoplasma gondii Hsp90 is a potential drug target whose expression and subcellular localization are developmentally regulated J Mol Biol 2005 350 4 723 734 10.1016/j.jmb.2005.05.031 15967463

[B44] Reniers J Robert S Frederick R Masereel B Vincent S Wouters J Synthesis and evaluation of beta-carboline derivatives as potential monoamine oxidase inhibitors Bioorg Med Chem 2011 19 1 134 144 10.1016/j.bmc.2010.11.041 21183355

