# Supplementary figures and images for: In vitro evaluation of β-carboline alkaloids as potential anti-Toxoplasma agents
Source: BMC Res Notes. 2013 May 10;6:193. doi: 10.1186/1756-0500-6-193 (PMC3654986; doi:10.1186/1756-0500-6-193)

## Slide 1
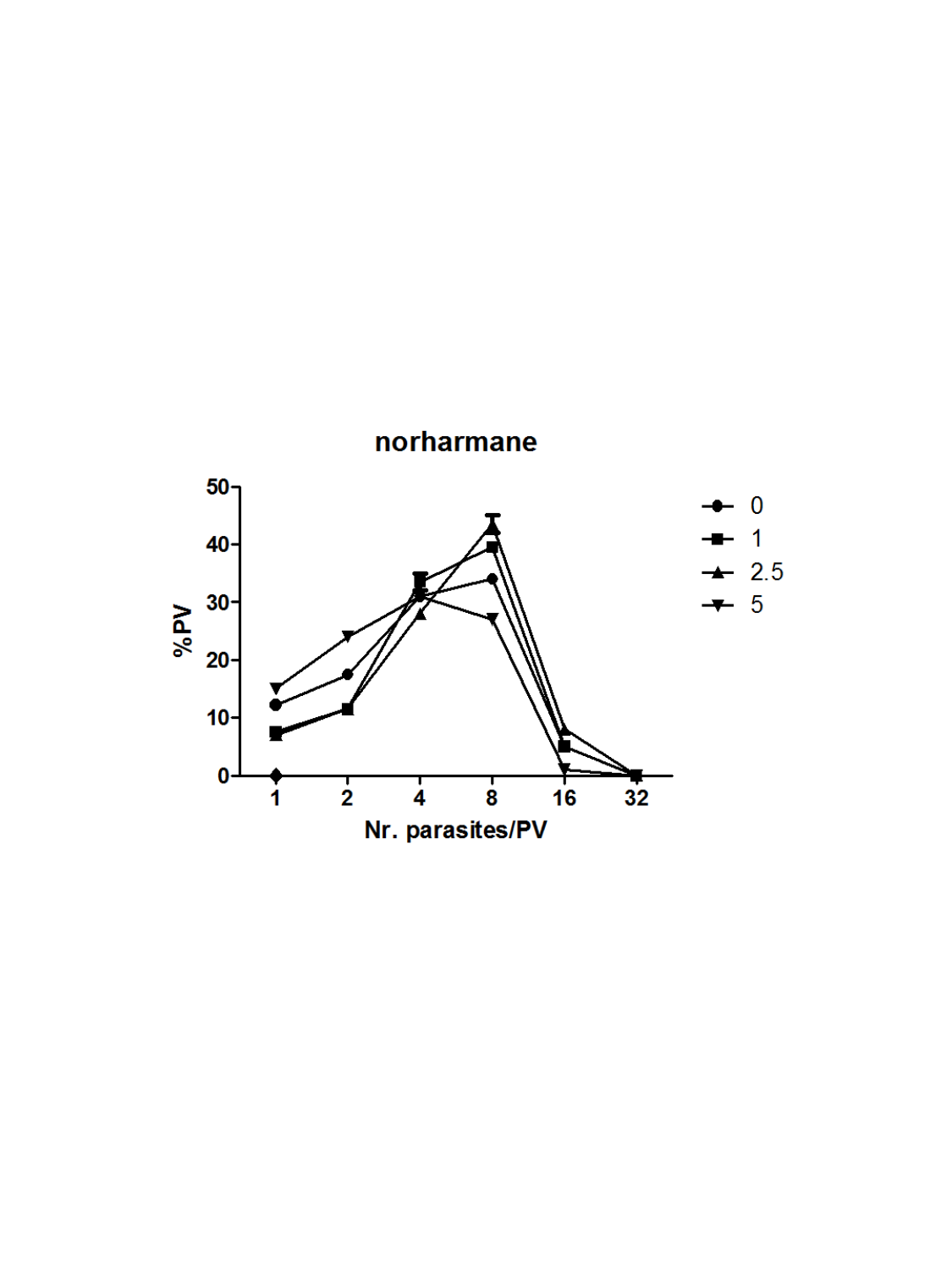

Supplement: Additional file 1: Figure S1 — Effect of norharmane on parasite invasion and replication. The analysis was similar to that mentioned in the Figure 3 legend, except that the drug doses were 0, 1, 2.5 and 5 μM. [file 1756-0500-6-193-S1.pptx]
